# Securing Your Airspace: Detection of Drones Trespassing Protected Areas

**DOI:** 10.3390/s24072028

**Published:** 2024-03-22

**Authors:** Alireza Famili, Angelos Stavrou, Haining Wang, Jung-Min (Jerry) Park, Ryan Gerdes

**Affiliations:** Department of Electrical and Computer Engineering, Virginia Tech, Arlington, VA 22203, USA; angelos@vt.edu (A.S.); hnw@vt.edu (H.W.); jungmin@vt.edu (J.-M.P.); rgerdes@vt.edu (R.G.)

**Keywords:** drone security, detection, classification

## Abstract

Unmanned Aerial Vehicle (UAV) deployment has risen rapidly in recent years. They are now used in a wide range of applications, from critical safety-of-life scenarios like nuclear power plant surveillance to entertainment and hobby applications. While the popularity of drones has grown lately, the associated intentional and unintentional security threats require adequate consideration. Thus, there is an urgent need for real-time accurate detection and classification of drones. This article provides an overview of drone detection approaches, highlighting their benefits and limitations. We analyze detection techniques that employ radars, acoustic and optical sensors, and emitted radio frequency (RF) signals. We compare their performance, accuracy, and cost under different operating conditions. We conclude that multi-sensor detection systems offer more compelling results, but further research is required.

## 1. Introduction

Unmanned Aerial Vehicles (UAVs) have evolved rapidly over the past few decades [1,2,3,4,5,6,7,8,9,10] leading to mass production of affordable drones [11,12]. From kids and hobbyists to police officers [13] and firefighters [14], drones have found novel applications and use cases [15,16,17,18,19,20,21,22,23,24]. For instance, Google and Amazon trialed drones for merchandise delivery while law enforcement leverages drones for speed checks [25,26,27,28,29,30]. During disasters, drones can help first responders establish communications and locate victims [31]. Unfortunately, drones can be used for illicit purposes, similar to other technological advancements [32]. Indeed, criminal groups use drones to smuggle goods and breach secure locations, to name a few. Even benign uses of drones can be unlawful, including unintentional invasion of privacy, harm to humans and infrastructure due to collisions, and interference with other flying objects (e.g., airplanes). For example, in 2016, Dubai airport reported that it had to shut down three times to avoid unauthorized drone activity [32].

**Figure 1:**
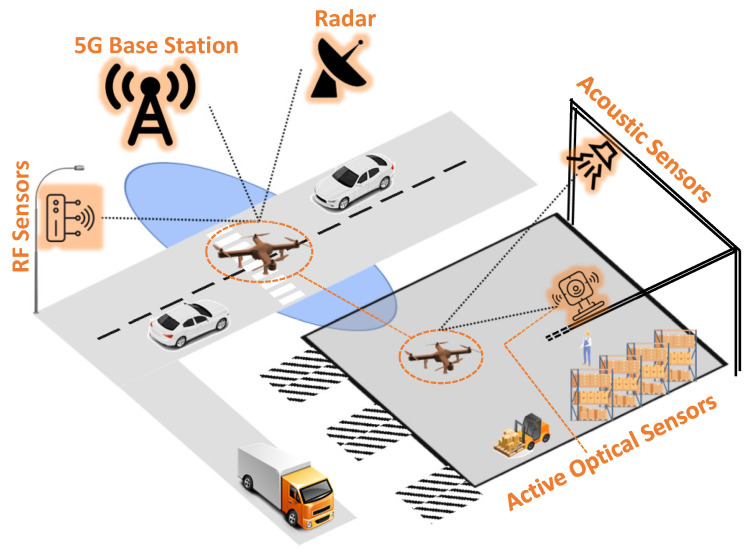
Different drone detection technologies.

Therefore, real-time drone detection, binary classification, and tracking have become a necessity [11,33,34]. The drone popularity, mixed use cases, and diverse environmental conditions have only exacerbated the detection challenges [35,36,37,38,39]. Currently, there are different methods for detecting drones in the airspace: active radars (e.g., [40]), passive radars (e.g., using spaceborne illuminators of opportunity [41]), acoustic sensors (e.g., [42]), Radio Frequency (RF) signal detection (e.g., [43]), and visual and optical sensors (e.g., [44]), as shown in Figure 1. The drone detection system is typically deployed in close proximity to the area of interest. When a drone enters a protected no-fly zone, the detection system can track it and determine whether it is a friendly or unknown intruder. Subsequently, the system can notify an operator or enforce an automated policy.

In this survey article, we present an overview of the available approaches for detecting drones. Our aim is to understand the design space for drone detection techniques and expose any inherent or situational limitations for each of these approaches. We also explore other aspects that are pertinent to selecting the drone detection approach, including cost, power consumption, accuracy, and environmental variables that might affect the performance of the detection system. We discuss radars more extensively as they are the most promising method in terms of accuracy. However, their high cost and deployment requirements can render radars unsuitable for some use cases. We then discuss off-the-shelf acoustic sensors as a cheaper but less accurate alternative to radars in some scenarios. Next, we explore approaches based on RF transmission of the drone followed by visual and optical sensor detection methods. Finally, we discuss multi-modal and sensor-fusion approaches. These use multiple sensors in tandem or sequentially to improve detection accuracy. Table 1 summarizes the advantages and disadvantages of the drone detection approaches discussed in this article.

## 2. Radars

The current state-of-the-art in moving object detection solutions, whether it is detecting a big drone or a small bird, involves some form of radar [45,46,47,48,49,50,51]. Radars offer high range coverage, uninterrupted operations in all weather environments, and continuous coverage during day and night. These capabilities have elevated radar technologies as one of the best candidates for drone detection systems [52,53,54,55,56,57,58]. Evaluating the feasibility of using radar for UAV detection has received considerable attention [40,59,60,61,62,63,64,65]. However, there are some practical limitations and cost considerations when designing and deploying a radar suitable for detecting drones.

### 2.1. Radar Configurations

**Figure 2:**
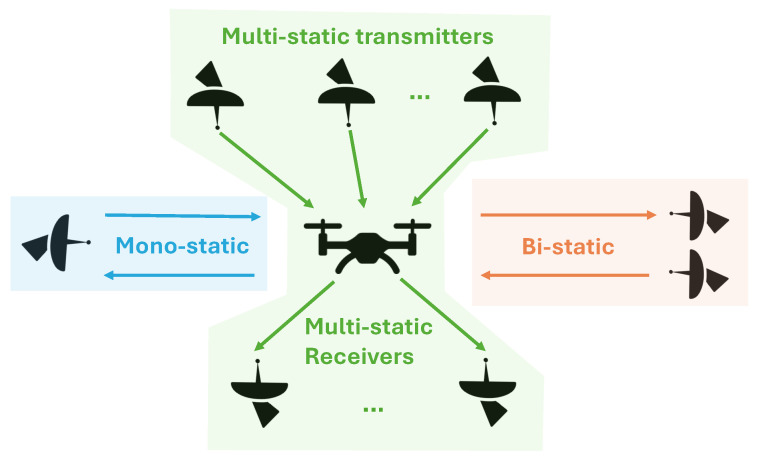
Visual representation of different radar configurations: Monostatic, Bistatic, and Multistatic.

Radar systems are categorized based on their configuration, particularly in terms of the spatial arrangement of their transmitter and receiver components. The three primary configurations are monostatic, bistatic, and multistatic, each with distinct characteristics, advantages, and applications [41]. A visual illustration of these three radar configurations is depicted in Figure 2.

#### 2.1.1. Monostatic Radar Configurations

In a monostatic radar setup, the transmitter and receiver are co-located or share the same antenna system. This configuration is the most familiar and widely used in various applications, from traffic enforcement to small drone detection [66,67,68,69,70]. The primary advantage of monostatic radar is its simplicity, as it requires only one site for both transmission and reception. This setup is highly efficient for short-range applications and where the target’s relative motion to the radar is significant, facilitating strong signal reflection back to the source.

#### 2.1.2. Bistatic Radar Configurations

Bistatic radars feature spatially separated transmitter and receiver sites, which can significantly vary in distance from each other. This separation introduces unique advantages, such as increased stealth since the receiver can be placed in a covert location away from the transmitter, reduced susceptibility to electronic countermeasures, and the ability to detect low-observable objects such as small UAVs. Bistatic radars are particularly advantageous in applications where monostatic radars are limited by reflection geometry or where the use of stealth is paramount [71,72,73,74,75]. However, the complexity of synchronizing the transmitter and receiver, along with the challenges in signal processing due to the geometry-dependent bistatic range, can complicate their operation.

#### 2.1.3. Multistatic Radar Configurations

Multistatic radars expand on the bistatic concept by utilizing multiple receivers and, in some cases, multiple transmitters [76]. This configuration offers enhanced coverage and detection capabilities, as multiple perspectives on the target can reveal its position and movement with greater accuracy and robustness against countermeasures [77,78,79,80,81]. Multistatic setups can effectively detect stealth aircraft, which are designed primarily to evade monostatic radars, by exploiting the varied angles of incidence and reflection captured by the dispersed receivers. The primary challenges in multistatic radar systems involve the complex coordination and data fusion from multiple sites, necessitating advanced signal processing and networking capabilities.

In general, the differences between these configurations lie mainly in the geometry of transmitter and receiver placements and the resulting operational advantages and complexities. Monostatic radars are simple and effective for a wide range of applications but can be limited by direct reflection requirements. Bistatic and multistatic radars, with their spatially diverse components, offer advantages in stealth detection and operational resilience at the cost of increased system complexity and signal processing requirements. More specifically, bistatic and multistatic radar configurations have enabled the capability for passive radar detection. This approach, which is not feasible in traditional monostatic setups, offers significant advantages by eliminating the need for active transmitters. This not only reduces costs and enhances stealth capabilities, making it harder for adversaries to detect the radar system, but it also circumvents the regulatory requirements for broadcasting signals. Passive radar systems leverage existing ambient electromagnetic emissions, such as those from television and radio broadcasts, cellular networks, and even satellite transmissions, to detect and track drones. By using these omnipresent signals, passive radars can effectively monitor airspace without the need for additional signal generation, blending cost efficiency with operational discretion. Further exploration of passive radar technology will be detailed in upcoming sections, highlighting its growing importance in modern surveillance and detection strategies.

### 2.2. Radar Cross Section

The main challenge is the variable size of UAVs, which can make them invisible to traditional radars. Due to the small size of some UAVs and their main body material construction, which can have a low reflection index, the Radar Cross Section (RCS) is extremely small and makes them hard to detect. As is shown in Equation (1), the received power from a target object is proportional to its RCS, a smaller RCS results in lower received power and a lower probability of detection [54].

(1) $$\begin{array}{c} P_{Rx}=P_{Tx}\cdot \frac{G_{Tx}\cdot G_{Rx}\cdot \lambda^{2}}{\left(4\pi^{3}\right)\cdot R^{4}}\cdot \sigma ; \end{array}$$

where $P_{Rx}$ is the target received power in the radar receiver, $P_{Tx}$ is the radar transmit power, $G_{Tx}$ is the radar transmitter gain, $G_{Rx}$ is the radar receiver gain, *R* is the range of the target, and σ is the RCS of the target.

### 2.3. Frequency and Bandwidth

Another design parameter for radars is their operating frequency. High-frequency radars are more expensive, but they can detect smaller-size UAVs [82]. Their larger bandwidth and finer resolution generate more accurate results. As is shown in Equation (2), the range resolution equals the speed of light divided by twice the bandwidth. As an example, a radar with a bandwidth of 1 GHz has a range resolution of 15 cm.

(2) $$\begin{array}{c} \Delta R=\frac{c}{2B}; \end{array}$$

where ΔR is the range resolution, *c* is the speed of light, and *B* is the radar bandwidth.

### 2.4. Radar Scattering

**Figure 3:**
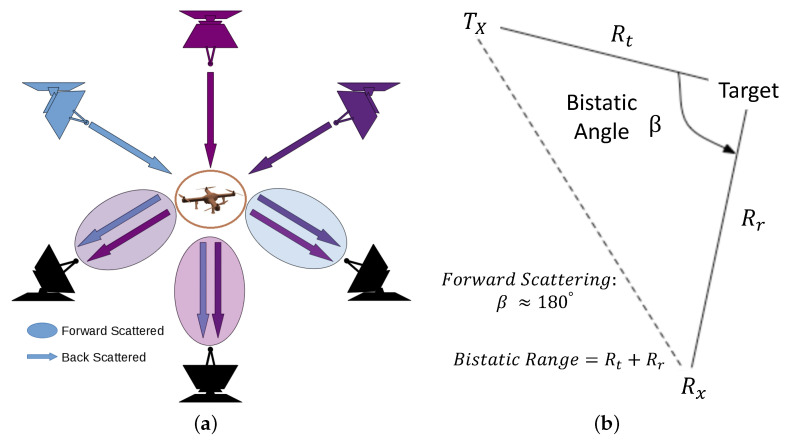
(**a**) Representation of backscattering vs. forward scattering radar configurations. Here, the colored radars depicted at the top of the figure represent the transmitters, while the black ones at the bottom represent the receivers; (**b**) Bistatic range and angle.

The physics behind radar systems encompasses two primary types of scattering: forward scattering and backscattering [41,76]. Forward scattering is detailed through a modern perspective, indicating that radar waves can scatter in the direction forward from their original path when the bistatic angle, the angle from the transmitter, through the target, to the receiver, is close to 180° [83,84,85,86]. This scattering is particularly advantageous for detecting small or RF-absorbent targets, such as small drones, due to the enhanced RCS in such conditions. Backscattering, on the other hand, refers to the reflection of radar waves directly back toward the receiver [87]. Figure 3a,b provide a visual illustration of the forward scattering/backscattering and the bistatic range/angle, respectively.

The RCS, a critical factor in drone detection, varies significantly with the target’s aspect angle, impacting detection capabilities. Different parts of a drone may have vastly different RCS values, influencing the effectiveness of radar detection from various angles. This variability underscores the benefit of multistatic radar systems, which utilize multiple receiver locations to increase the likelihood of detecting backscattered signals from various aspect angles of a target.

Forward scattering offers a distinct advantage by enhancing detection capabilities for targets that are otherwise challenging to detect due to size or material properties. However, maintaining an optimal bistatic angle for forward scattering can be challenging over time, limiting the practicality of such systems in some scenarios. Despite these challenges, forward scattering radars, especially in multistatic configurations with spaceborne illuminators, are gaining renewed interest for their potential in broad-area surveillance, demonstrating high detection probabilities even when considering real-world losses.

### 2.5. Radar Signal Power

In practice, radars with higher transmission power offer improved detection results. In terms of wave modulation, CW (Continuous Wave) radars require significantly less power than pulsed versions [40]. Thus, CW radars are more attractive for small UAV detection. Many of the available research papers (e.g., [40]) use FMCW (Frequency Modulated Continuous Wave) radars for drone detection. This is due to their lower power consumption compared to other wave modulation approaches.

### 2.6. Active or Passive

There are two different types of radars: active and passive. Active radars are equipped with both a transmitter and a receiver. The transmitter emits electromagnetic waves, which illuminate proximal targets. The receiver captures all reflected signals, which are then post-processed to expose any potential new targets. When only passive sensing is employed, the radar system is reduced to only receivers. Target illumination in the passive radar scenario is done by other signal sources, including cellular signals, FM radio signals, and Wi-Fi signals, among others. As an example, in [61], Chadwick demonstrated the feasibility of micro-drone detection at ground level using a software-defined radio receiver and UMTS 3G signals as a source of illumination. An additional illustration can be found in the work of Robie et al. [76], wherein they presented a conceptual framework for assessing the probability of signal interception through a model that gauges the received signal power and coverage from satellite illumination at ground level. To expand the geographical scope and detection potential in comparison to contemporary methodologies, they recommended leveraging the available spaceborne illuminators, such as the proliferated Low Earth Orbit (LEO) telecommunications satellite constellations like Starlink. Their research showcases the fact that these constellations work in the Ka-band, resulting in significant target RCS values when utilizing a forward scattering radar configuration.

Active sensing achieves a higher range of detection and better reliability, but it requires significantly more transmit power. Moreover, active sensing might not be capable of illuminating targets under diverse environmental conditions. Also, the radar operator needs to obtain a license and maintain permits for the band that the radar transmitter signal occupies. On the other hand, passive radars do not require any operational permits because they do not actively transmit signals. Furthermore, their power consumption and cost requirements are significantly lower. Therefore, an operator can accommodate multiple receivers for the same budget of a single active radar deployment. For instance, Chadwick et al. [61] proposed a system for drone detection using passive radar technology leveraging available UMTS 3G cellular communication signals as illumination sources. They considered three different ways to illuminate: using a cell phone on a call, having micro base stations for 3G communication, and using the base stations in the closest vicinity. All three options were deployed in the target area. They use two receivers. One is for capturing the genuine signal before all the reflections, and the other receiver is responsible for obtaining reflected signals. While this passive radar solution is cost-effective, it comes at the expense of accuracy and lack of reliable coverage.

### 2.7. Beam Steering

The more focused and narrow the transmitted signal, the better the illumination for detecting small objects. For instance, using omnidirectional antennas with a wide main lobe will result in poor performance in detecting small objects. On the other hand, using a narrow radar beam with a focused main lobe, while accurate for small objects, it decreases the surveillance perimeter. One option is to use several antennas on the transmitter side. Each antenna has a narrow beam but is placed in such a formation that, combined, they cover the target area. Another option is to make the transmitter mobile by using a rotor. This method is called mechanical beam steering, and it can cover the target area over a period of time. In addition to mechanical beam steering, there is another approach called electrical beam steering, in which a narrow transmitted beam scans small areas by changing the phase of the signal over time, resulting in full target area coverage.

### 2.8. Mechanical or Multi-Channel Scanning

The receiver can also be installed on a motor that can mechanically turn and scan the whole area. An alternative design makes use of static multi-channel antennas that can receive signals from any direction. Most active radar scenarios where the transmitter and receiver are bundled together are usually either static multi-channel or use a mechanical rotation for both reception and transmission of signals. As an example, in [40], Noetel et al. investigate two methods of scanning. In the first scenario, they used a scanning surveillance radar system. This is a mechanically scanning FMCW system operating in 94 GHz (they used mmWave radar). This radar can scan using 8 Hz frequency, resulting in an image update rate of 8 frames per second. In the second scenario, they used a static multi-channel radar. The radar was equipped with four channels on the receiver side to cover the whole area. It was also able to determine the 3D location of the target. The multi-channel approach can be used in situations where mechanical scanning is prohibited. In both of the scenarios, since they used FMCW radar, the power consumption is low. In addition, they were able to achieve good visibility of small objects and range resolution of 15 cm. This was due to the 1 GHz bandwidth supported by the mmWave radar.

### 2.9. Micro-Doppler Analysis

**Figure 4:**
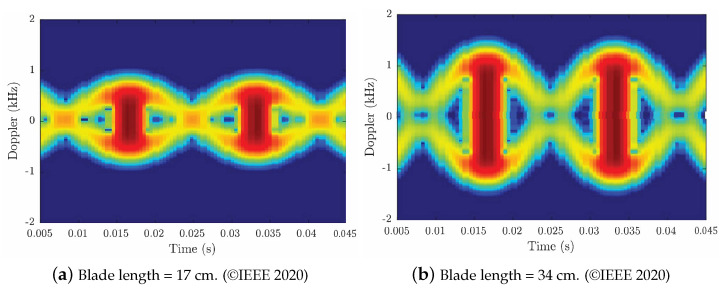
Micro−Doppler analysis simulation: (**a**) Two 17 cm blades (**b**) Two 34 cm blades, rotating at 30 Hz RPM which captured by a CW radar with center frequency of 2.41 GHz. This figure is a replica of the one presented in the work of Gannon and Tahmoush as illustrated in [90].

Micro-Doppler analysis is used in radar analysis to fingerprint and identify target objects [88]. This is different than the Doppler effect used to determine the speed and direction of the target object. Any vibration or movement in the target object’s body or any other moving parts onboard the target can be measured using micro-Doppler analysis [89]. When analyzing the reflected radar signal from drones, the primary source of making micro-Doppler analysis feasible is the drone propellers. Micro-Doppler analysis can assist in distinguishing between drones and birds, thereby reducing false alarms. Additionally, using micro-Doppler analysis, we can estimate the structural features of the target drone. This includes the length of the rotors’ blades [90]. For example, in Figure 4, Gannon et al. [90] illustrated that when the size of a propeller’s blades increases from 17 cm to 34 cm, the Doppler response is doubled. This experiment assumed that the drone maintained the same rotation frequency of 30 Hz.

### 2.10. Future Radar Drone Detection

One promising research direction is to leverage commodity 5G cellular communications for drone detection. Terrestrial and satellite 5G communications can be used as both passive or active radar sources to illuminate and detect drones. Numerous research studies aim to investigate the challenges and limitations of harnessing existing and future 5G infrastructure capabilities for drone detection. As an example, Solomitckii et al. [59] explored the idea of using 5G base station antennas for drone detection. Since 5G can employ mmWave antennas in the base station for communications, it is conceivable that the 5G infrastructure can also be used as radar for detection purposes. In addition, Wang et al. [60] presented successful experimental results for drone detection from antennas operating in 28 GHz. These frequencies are similar to the frequencies 5G base station antennas use.

We have discussed the challenges and potential design parameters when selecting an appropriate radar for detecting small UAVs. We want to reiterate that there are many parameters to consider. The size and materials of the target UAVs, operating environment limitations, and the type of radar systems used are just some of the primary solution drivers. We also need to take into account the associated cost of operation and deployment. In the following sections, we demonstrate how radar sensors can be used alone or in combination with other sensors to increase the reliability of flying object detection.

## 3. Acoustic Sensors

Drone detection using acoustic signals is emerging as a pivotal technique in the realm of security and surveillance, leveraging the unique noise signatures generated by drones. This method capitalizes on the distinct acoustic patterns produced by drone propellers and motors, allowing for the identification and tracking of drones even in visually obstructed environments. Advanced signal processing and deep learning techniques are increasingly being employed to enhance the accuracy and speed of detection, making this approach highly effective in safeguarding privacy, ensuring security, and monitoring restricted spaces. The sophistication of acoustic sensor technology, coupled with the integration of AI-driven analysis, paves the way for real-time, reliable drone surveillance systems. Some examples of the available research and literature that focus on drone detection using acoustic sensors can be found in [42,91,92,93,94,95].

As an example, in [91], Dumitrescu et al. focused on the development of an acoustic system for UAV detection. More specifically, they focused on the creation and application of an advanced acoustic system aimed at identifying, locating, and communicating the position of UAVs. The core of the proposed detection and location mechanism relies on the analysis of acoustic signals and the application of concurrent neural networks (CoNNs). They detailed the development of software functional components integral to their detection and location algorithm. Further, they elaborated on the evaluation of detection and tracking effectiveness for remotely piloted aircraft systems (RPASs) utilizing a specialized spiral microphone array equipped with Micro-Electro-Mechanical System (MEMS) microphones. The detection and tracking algorithms were formulated through the decomposition of spectrograms and the use of adaptive filters. Notably, their research utilized various techniques such as Cohen class decomposition of spectrograms, log-Mel spectrograms, harmonic-percussive source separation, and analysis of raw audio waveforms collected from the spiral microphone array. These methodologies were employed to feed CoNNs, enabling the precise identification and classification of drones within the monitored perimeter.

While economical, acoustic sensors have some significant drawbacks that need to be considered when it comes to drone detection. Their primary limitation is that their performance is highly dependent on the target’s range (distance) from the sensor. The maximum range provided by state-of-the-art acoustic detection mechanisms is in the order of a few hundred meters. To make matters worse, in crowded and noisy urban environments polluted with ambient sounds and noise, the performance of acoustic sensors degrades drastically. Thus, acoustic sensors perform poorly in detection scenarios where patrol drones or other noisy equipment are employed to conduct the surveillance. In general, in any deployment scenario where the ambient noise is too high, acoustic sensors perform poorly. On the plus side, acoustic sensors are inexpensive and can be easily acquired, installed, and deployed. In addition, they can perform well under any weather conditions, both in the daytime and at night, and they do not need a Line of Sight (LoS) to the target object. All being said, when used on their own, they do not offer performance guarantees due to their aforementioned drawbacks. However, as a companion sensor, they can boost the overall system performance and accuracy.

Furthermore, the application of deep learning techniques to drone detection and identification using acoustic features is gaining prominence due to the increasing utilization of drones in various sectors and the associated security concerns. A novel approach has been developed by Al-Emadi et al. in [93] that automates the process of drone detection and identification by harnessing the acoustic characteristics of drones. This method leverages different deep learning algorithms, addressing the challenge posed by the scarcity of acoustic drone datasets. A hybrid drone acoustic dataset has been created, combining recorded drone audio clips and artificially generated drone audio samples using Generative Adversarial Networks (GANs). The effectiveness of drone audio in conjunction with deep learning algorithms such as Convolutional Neural Networks (CNNs), Recurrent Neural Networks (RNNs), and Convolutional Recurrent Neural Networks (CRNNs) in drone detection and identification has been explored. The study confirms the advantages of applying deep learning techniques to this domain and highlights the beneficial role of GANs in generating realistic drone audio clips, enhancing the system’s capability to detect new and unfamiliar drones.

Expanding the horizons of acoustic surveillance, the deployment of acoustic sensors atop UAVs opens a new realm of possibilities, including patrolling and monitoring roles. These drone-mounted acoustic cameras can serve as vigilant sentinels in the sky, not only for detecting other drones but also for a broader range of surveillance applications. In this context, we can refer to the work of Salom et al. [94]. They introduced an acoustic camera intended for direct attachment to a UAV airship. Comprising 64 microphones, a central processing unit, and software explicitly devised for detecting low-level acoustic signals in the far field, this innovation is a testament to the evolving capabilities of aerial surveillance. With an aperture spanning 2 m and designed for operations at altitudes up to 300 m, the camera is a tool for a spectrum of applications, such as urban and industrial noise monitoring, security surveillance, rescue operations, and wildlife monitoring. Although their initial demonstrations were ground-based, the camera is poised for airship integration, marking an example in the domain of acoustic surveillance from the skies.

## 4. RF Ground Communication Sensors

One of the most widely used approaches to detect the presence of a drone in no-fly zones is by sensing the RF communications between the drone and ground controllers [96]. This method leverages RF sensors working as receivers scanning for RF communication channel transmissions [96,97,98,99,100,101,102]. The RF sensors are designed to detect the RF frequency ranges that drones use for control and data signaling with ground controllers. The first step is to distinguish existing versus new RF communications. Then, using the newly extracted RF communications, they have to further identify unique RF signatures for drones using techniques such as the ones presented in [32,43,103,104,105].

As an example, in [104], Nemer et al. presented a UAV identification and detection system based on a hierarchical concept using ensemble learning. Their system had four classifiers that worked in a hierarchical fashion. The first classifier determines whether the UAV is available, and the second one specifies the type of UAV detected. The last two classifiers specify the flying mode of some UAVs (merely on, hovering, flying, flying with video recording). They had a pre-processing stage with feature extraction and noise filtering to improve performance.

In another example [99], Alam et al. discussed a comprehensive system combining RF signals and deep learning for detecting and identifying drones. Their approach involves an advanced multiscale convolutional network model to process the RF data. They validated the effectiveness of their model through various metrics, demonstrating high accuracy in distinguishing different drones and identifying specific models, even in challenging conditions with noise.

Digulescu et al. explored using Ultra-Wideband (UWB) sensing to distinguish between the drone and human movements indoors [106]. They employed advanced signal processing methods like wavelet transform and phase diagram concepts to process UWB sensor data, aiming to enhance security by differentiating between authorized human presence and potential drone threats in sensitive areas.

For RF sensing of drones, the common assumption across all approaches is that there exists an RF communication link between the target drone and its ground controller. It is further assumed that this control signal can be captured and precisely analyzed even in the presence of other signals. Indeed, for many commercial drones, RF signals are the primary means for communicating navigation commands to the drone from the ground controllers and, reversely, when downloading captured data such as images, videos, and other sensory information captured by the drones. While these assumptions are valid for many commercial off-the-shelf drones, there are drones capable of flying autonomously without the need to receive periodic navigation commands. Moreover, in some scenarios, drones are equipped with an adequate amount of onboard memory to capture sensory information for prolonged periods of time. Thus, even when a drone supports RF communications, there could be extended periods of time in which there is no RF communication between drones and ground controllers. Another challenge with RF sensing for drone detection is the presence of environmental RF noise. This is especially true in urban areas where wireless activity is prevalent, generating overlapping and constant RF transmissions emanating from both ground and aerial targets that are not necessarily drones. For instance, people use their Wi-Fi devices to stream videos from the Internet while they are walking on the high floors of a tall building, which resembles the movement and transmission originating from a drone. Thus, merely depending on RF sensing is not reliable for urban environments due to environmental and noise considerations, including the presence of multiple concurrent communications from both stationary and moving targets. On the other hand, in less populated or rural areas where there are few wireless devices, the RF channels are primarily silent. Therefore, it is easy to sense the communications between drones and their ground controller.

**Figure 5:**
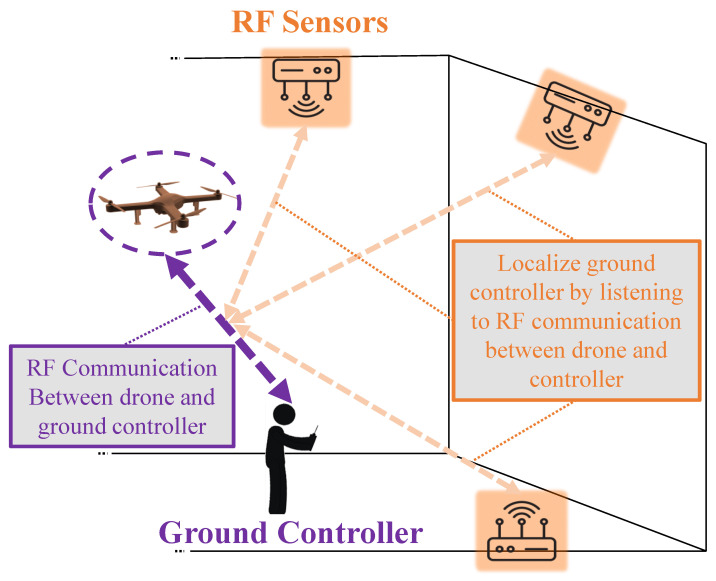
Using RF sensors to localize the ground controller of the intruder drone.

While using RF ground communication signals for drone detection has limitations, it offers a cost-efficient and easy-to-implement mechanism [43,107,108,109,110,111,112]. It can be useful when operating over a more extended area and period of time. Moreover, it can be combined with other sensors as it operates under any weather or light conditions, and it does not need direct LoS. Additionally, this method can detect the drone even before it takes off and when it appears to be stationary (i.e., the drone has landed or it is just hovering). As long as there exists an active RF communication link from or to the drone, the RF sensors can detect it [113,114,115]. More importantly, this is the only method that can locate the ground controller of the drone as well as the drone itself [116,117]. In other words, among the various drone detection methodologies, the utilization of RF signals stands out due to its unique capability not only to detect drones but also to pinpoint the location of the drone controller on the ground using various positioning techniques [118,119,120,121,122,123,124]. To achieve this, it leverages the communication link between the drone and its controller, which continuously exchanges RF signals for operation and control. By analyzing these signals, it is possible to trace back to the controller’s exact location, providing a significant advantage in scenarios where identifying the operator is as critical as detecting the drone itself. This dual-functionality aspect of RF signal analysis makes it a critical tool in comprehensive drone surveillance and security measures, offering a layer of intelligence that purely detection-oriented techniques cannot provide. A graphic representation of RF sensing for drone detection and localizing the controller is depicted in Figure 5.

## 5. Optical Sensors

Optical sensors include cameras, gated lasers, and other visual sensing modalities that perform optical processing. The use of optical sensors provides another approach to detect and classify UAVs [125]. Similar to using radars, there are two approaches for deploying optical sensors: active and passive. In active sensing, the detection system leverages an optical signal emitted by a gated laser (e.g., LiDAR [126]) to illuminate an area or a target of interest. The detection occurs by processing the reflected optical signals from the target. The passive method leverages an optical receptor such as a camera to capture images or video for visual processing and classification of drones. The main advantage of using cameras is their ability to reveal additional information assisting drone classification. Image and video processing techniques can be applied to distinguish between drones and other flying objects or birds. Visual classification can separate intruder and friendly drones and determine the type of drone. Thus, optical sensing can go beyond mere object detection to object classification with high accuracy when available.

As an example of active optical systems, in [127], Chen et al. explored the application of LiDAR in drone detection, particularly focusing on the challenges posed by small UAVs and their detection at long distances due to low laser energy reflection. This research highlights the potential of single-photon LiDAR systems, known for their high sensitivity and temporal resolution, to detect UAVs even in night environments. The system utilizes time-correlated single photon counting (TCSPC) for high-resolution drone search, emphasizing the influence of the field of view (FOV) on detection efficacy, hence representing a step toward practical, low-power drone detection.

Furthermore, in another study [128] proposed by Aldao et al., a detect and avoid system for UAV navigation in Urban Air Mobility (UAM) corridors is introduced, utilizing a solid-state LiDAR sensor for detecting and positioning unauthorized flying objects within corridor airspace. Their proposed system, leveraging point clouds generated by the sensor and a Second Order Cone Program (SOCP), computes real-time avoidance trajectories. They provided tests in various scenarios and showed results with execution times suitable for real-time implementation on modern onboard computers.

In [129], as an example study of image-based passive optical systems, Lv et al. presented a method to improve drone detection accuracy and speed in high-resolution images using a combination of background difference and a lightweight network. The approach includes advanced features like the Ghost module and Simplified Attention Module (SimAM) attention mechanism to enhance feature extraction and accuracy. The Ghost module is a neural network design that aims to reduce computational requirements by generating more feature maps from cheaper operations. SimAM is an attention mechanism designed to enhance the representational capacity of convolutional neural networks by recalibrating feature maps in a computationally efficient manner. Both are innovations intended to optimize performance and efficiency in deep learning models, such as those used for drone detection. Their method achieved some improvements in detection accuracy and speed, balancing efficiency and precision for high-resolution drone detection.

**Figure 6:**
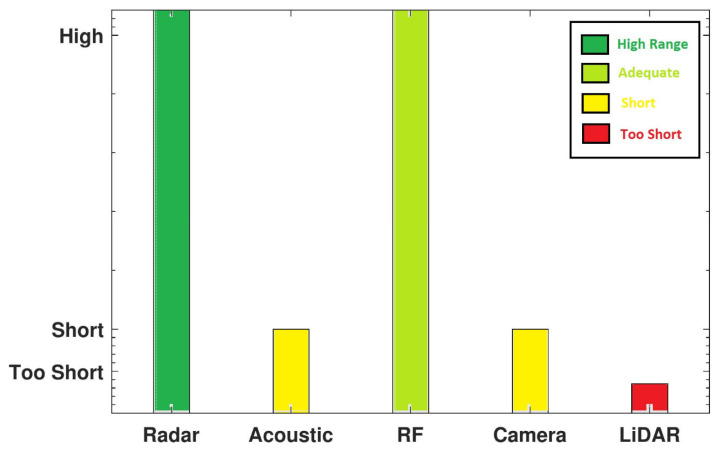
Maximum range of detection for different sensors. ‘High’ represents ranges around 1 mile, ‘Short’ is for less than 1000 feet, and ‘Too Short’ indicates less than 350 feet.

The major drawback of optical sensors is their dependence on an uninhibited LoS to the target. Moreover, their accuracy degrades significantly in visually impaired environments. For instance, even when using night vision cameras, the quality of captured information in reduced or deprived light settings is far from optimal. In fact, cameras may fail to produce reliable detection results for small targets under different weather conditions (e.g., foggy, cloudy, rainy, etc). Another limitation is that cameras offer a narrow beam for detection. This means that single cameras cannot cover large areas of interest at once. Therefore, we have to use multiple cameras or rotate one camera to swipe the area of interest. While active visual sensing (i.e., lasers) are not as sensitive as regular cameras to weather conditions, they can only provide detection at a very short range from the target. A graphic representation of the maximum range of detection for different sensors is shown in Figure 6. Hammer et al. [44] conducted experimental tests to evaluate the feasibility and practical performance of employing LiDAR for drone detection systems. While the results appear to be encouraging, the system had to operate in a very short range, requiring a direct LoS to the target. When the target was within the sensor LoS and at a short range to the LiDAR system, a full 3D scan of the target was produced.

## 6. Multi-Sensor Approach

All of the sensor modalities have limitations that can render them unreliable under certain environmental and weather conditions [130,131]. We posit that a robust drone detection system should rely on more than one sensing modality. Aptly chosen sensing modalities can complement each other and increase the overall reliability and identification robustness. Therefore, we can achieve better performance by fusing different types of sensors based on environmental conditions.

In other words, sensors need to complement each other’s shortcomings to enhance the overall system quality and reduce the risk of misdetection. For instance, an acoustic system alone may perform poorly because it cannot detect drones at higher altitudes. However, by integrating this system with active radar, we can achieve detection over longer ranges. Furthermore, we can improve the accuracy of detection for short ranges by designing a system that fuses data from both radar and acoustic sensors. For long ranges, we assign a much higher weight to the radar’s output, and for shorter ranges, we assign greater weight to the acoustic sensors. This approach ensures a better quality of detection across all ranges. In other words, when combining results from different sensors, a system needs to assign weight to the outcomes based on their strengths and shortcomings. This way, at any given time, we trust the sensor that has better strength for that specific scenario, while in situations where that sensor performs poorly, the other sensors will have a higher weight in the decision-making process and complement the system.

To that end, Laurenzis et al. [132] collected data from a heterogeneous sensor network consisting of acoustic antennas, small FMCW radar systems, and optical sensors. The authors applied acoustic sensors, radar, and LiDAR. Their goal was to monitor a wide azimuthal area (360 degree) to simultaneously track multiple drones with various degrees of success. In addition, they deployed optical sensors for sequential identification with a very narrow field of view. In another example [133], Giovanneschi et al. propose a drone detection system that consists of two stations. One was a static multi-sensory network, and the other one was a sensor unit installed onboard a moving vehicle. They initially studied a fixed multi-sensory network that included an acoustic antenna array, a stationary FMCW radar, and a passive/active optical sensor unit. The active optical sensor was LiDAR. A mobile vehicle equipped with passive/active optical sensing was brought in to augment the sensory network and cover areas behind obstacles. In contrast, the static multi-sensory network monitored a stationary area with a sensor-dependent sensing coverage. The data fusion from the multi-sensory network and the moving vehicle offered better performance for target detection.

## 7. Discussion

After a comprehensive investigation of existing research, it was concluded that a reliable drone detection system requires a combination of multiple sensor modalities. In light of this, we present a few sample systems that we have designed and proposed for further investigation and performance evaluation in future research.

**Figure 7:**
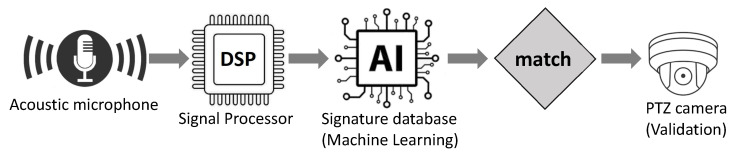
Acoustic antenna arrays for drone detection used in conjunction with a PTZ camera to confirm the presence of the drone and classify it as benign or intruder.

Figure 7 portrays the initial exemplar that we have devised as a multi-sensor drone detection system. As is shown in the figure, first, an off-the-shelf low-energy acoustic sensor captures all acoustic signals in the environment and uses machine learning algorithms to process the signals. If the algorithms detect the presence of a drone, it triggers another sensor where a pan tilt zoom (PTZ) camera takes the stage to confirm the detection and classify the drone as friendly or intruder.

As an additional example of a detection system, we suggest utilizing 5G cell towers in an innovative adaptive multi-sensor system to identify the existence of an intruder drone, classify its type, and locate its ground controller. The massive antenna-array systems in 5G cell towers have the potential to be utilized as high-frequency, high-resolution radars suitable for detecting drones, as has been demonstrated in prior research [134,135,136].

In this proposed example, an adaptive multi-sensor detection system is presented that combines 5G technology with additional auxiliary sensing modalities to address scenarios where 5G has limitations. The system is designed to operate in both crowded urban environments and quiet rural areas and consists of three main blocks: Detection, Validation, and Localization. These blocks work together to provide drone detection, classification, and localization of the ground controller. Further details on each block will be provided, including the key role played by 5G technology in achieving the system’s objectives.

**Detection:** The first step in the drone detection process is the realization of the presence of any airborne object, including friendly or hostile drones. In this part, we show the detection block and explain how it can adapt to different environments. The 5G base station antennas, operating as active radars with millimeter-wave technology, and acoustic antenna arrays are the two main components of this block.

While radars are the most promising method for detecting airborne objects—especially in crowded urban areas with visual impairment and RF noise—using conventional radars is challenging due to the small size of commercial drones. However, by employing dense 5G base station networks in the urban area, the system benefits from the high-frequency mmWave signals, which are better suited for detecting small objects. The radar’s transmitted signal must be shorter than the object’s size to detect it. Higher frequencies available in 5G mmWave technology means smaller wavelengths and better visibility, even for small drones. Moreover, the large bandwidth available in the 5G mmWave range increases the resolution. This was explained in detail in Equation (2).

The phased antenna array in the 5G base station provides an electronic scanning capability, making the process more reliable and faster than mechanically scanning dishes. Additionally, using the existing 5G infrastructure offers cost-efficiency and avoids the need to install additional high-frequency radars. Finally, the received signals from 5G base station antennas can undergo micro-Doppler analysis, allowing for more precise information on the detected object’s shape, type, and other features.

The complementary sensors employed in the detection block are the acoustic antenna array receivers, which can perceive the propeller noise of the drone. By utilizing a pre-trained machine learning model using the acoustic signals obtained by the acoustic antenna arrays in different environments and drone scenarios, we can accurately detect the drone’s presence in the environment. The acoustic sensor is cost-effective, both in terms of equipment and power consumption, but it has limitations in terms of short ranges and poor performance in noisy environments. This sensor can be valuable in scenarios where noise is minimal, such as in rural areas where the density of 5G base stations is insufficient to establish a reliable drone detection system.

In summary, the core of our drone detection system is the detection block, which encompasses two sensors. Firstly, we employ 5G base station antennas functioning as high-frequency large bandwidth radars that can track an intruding drone by beam-steering through its Multiple-Input Multiple-Output (MIMO) technology. Secondly, we utilize an acoustic antenna array connected to a machine learning program that can detect the presence and the type of drone by analyzing the propeller noise received by the array. The data obtained from these two sensors are fused together in a decision algorithm, where each datum has a unique weight based on the environmental conditions. For instance, in a densely populated urban area, more emphasis is given to the 5G data, whereas in rural areas with fewer 5G base stations, the system assigns greater weight to the data acquired from the acoustic sensors. Thus, the system overcomes the limitations of each sensor by compensating for each other’s flaws, providing a reliable drone detection mechanism.

**Validation:** The aim of this stage is twofold: to verify the identified object with greater scrutiny and to classify it accurately by eliminating the likelihood of falsely detecting small entities such as birds or friendly drones. This is accomplished by directing a camera towards the identified target. While in less crowded surroundings, such as rural areas, a PTZ camera with remote directional and zoom capability would suffice, in crowded urban settings, where multiple obstacles obstruct the view, the system harnesses a surveillance drone equipped with a camera, which can approach the target for a closer look. To facilitate communication between the surveillance drone and the ground controller, such as navigating the surveillance drone toward the target and transmitting real-time video data, 5G technology’s sidelink communications are employed.

**Localization of the Controller:** Up to this point, we have outlined the initial two blocks of our drone detection process. Here, we introduce the use of an RF sensor, which is utilized in both rural and urban environments, to locate the controller of the drone on the ground, which is crucial for subsequent steps in the detection process. With this, we conclude our proposed multi-sensor drone detection system, which employs a fusion of various sensor modalities working in tandem to ensure a robust drone detection system. A summary of our adaptive multi-sensory drone detection methodology can be found in Table 2.

## 8. Conclusions & Future Work Discussion

We presented an overview of the available methods for drone detection. The radar sensors appear to be the most promising approach for detecting drones. However, their cost is relatively high. On the other hand, acoustic sensors are limited to low-noise environments but offer low energy and deployment costs. Furthermore, we discussed how RF sensing can detect the drone’s communications with a ground controller. However, many drones can fly autonomously and remain silent for a prolonged period of time. This will hinder RF sensing from detecting their presence. We also discussed optical sensors that can be used actively, such as LiDAR, or in passive mode, like video and still imaging. Visual sensors offer advantages when it comes to target identification. However, their accuracy is impaired by distance, lack of LoS to the target, and environmental conditions. Finally, we presented recent studies that combine different sensing modalities to develop more reliable and accurate approaches for drone detection.

Our survey clearly indicates that using multiple classes of sensors can mitigate some of the individual sensor limitations. Moreover, it can boost detection robustness under adverse operational scenarios. There are clear trade-offs between energy consumption, cost, performance, and operational requirements that individual sensors might fail to optimize. The use of multiple sensing modalities that are operational only when needed might be the answer. Thus, improving multi-sensor performance using combined cross-sensing learning algorithms and on-demand versus continuous sensing should be investigated further in future studies.

## Figures and Tables

**Table 1:** Comparison of drone detection approaches.

Detection Techniques	Advantages	Disadvantages
**Radar**	Operates in day/night, acoustically noisy, and visually impaired environments. Long range. Constant tracking. Performs even if drone flies autonomously (without RF emissions). Drone size/type detection via micro-Doppler analysis.	Small RCS can affect performance (in cmWave radars, for regular-sized commercial drones, RCS typically ranges from −1 dBsm to −18 dBsm). Active radars require transmission license and frequency check to prevent interference.
**Acoustic microphone array**	Operates independent of visual conditions (day, night, fog, etc.). Performs even if drone flies autonomously. Operates in LoS/NLoS. Low-cost implementation. Low energy consumption. Easy to deploy.	Short detection range (detection range < 500 m). Performance degrades in loud and noisy environments. May work poorly in crowded urban environments due to acoustic noise.
**RF signals**	Operates in day/night, acoustically noisy, visually impaired, and LoS/NLoS environments. No licence required. Low cost sensors (e.g., SDRs). Can locate the controller of the drone on the ground.	Detection fails in cases where there is no RF signal transmission from the drone. May work poorly in crowded urban areas due to RF interference.
**Vision-based**	Offers ancillary information to classify the exact type of drone. Can record images as forensic evidence for use in eventual prosecution.	Short detection range (e.g., LiDAR sensors’ range < 50 m). Requires LoS. Relative expensive sensors. High computational cost. Performance degrades under different weather conditions (fog, dust, clouds, etc) and in visually impaired environments.
**Multi-sensor**	Combines advantages of multiple methods. Has better overall performance. Robust under different scenarios and environmental conditions.	Increased cost and computational complexity compared to single sensors.

**Table 2:** Our proposed adaptive multi-sensor detection system.

Stations	Detection	Validation	Localization
**Urban**	The 5G cell tower antennas function as phased-array active radars with high-resolution	Surveillance drones equipped with cameras (optical sensors) that can fly close to the target and provide a better view	RF sensing to localize the controller of drone on the ground
**Rural**	Machine learning program to analyze data captured with acoustic antenna arrays	PTZ camera to provide a better view at the target	RF sensing to localize the controller of drone on the ground

## Data Availability

All data utilized in this study were generated using MATLAB 2021b software. The data are available for sharing upon individual request.

## Abbreviations

The following abbreviations are used in this manuscript:

UAV	Unmanned Aerial Vehicle
RF	Radio Frequency
RCS	Radar Cross Section
CW	Continuous Wave
FMCW	Frequency Modulated Continuous Wave
LoS	Line of Sight
PTZ	Pan Tilt Zoom
MIMO	Multiple-Input Multiple-Output
GAN	Generative Adversarial Network
CNN	Convolutional Neural Networks
RNN	Recurrent Neural Network
CRNN	Convolutional Recurrent Neural Network
SimAM	Simplified Attention Module
UWB	Ultra-Wideband
TCSPC	Time-Correlated Single Photon Counting
FOV	Field of View
UAM	Urban Air Mobility
SOCP	Second Order Cone Program
CoNNs	Concurrent Neural Networks
MEMS	Micro-Electro-Mechanical Systems
RPASs	Remotely Piloted Aircraft Systems

## References

[1] Choi H.W., Kim H.J., Kim S.K., Na W.S. (2023). An Overview of Drone Applications in the Construction Industry. Drones.

[2] Quamar M.M., Al-Ramadan B., Khan K., Shafiullah M., El Ferik S. (2023). Advancements and Applications of Drone-Integrated Geographic Information System Technology–A Review. Remote Sens..

[3] Raivi A.M., Huda S.M.A., Alam M.M., Moh S. (2023). Drone Routing for Drone-Based Delivery Systems: A Review of Trajectory Planning, Charging, and Security. Sensors.

[4] Famili A., Park J.M.J. ROLATIN: Robust Localization and Tracking for Indoor Navigation of Drones. Proceedings of the 2020 IEEE Wireless Communications and Networking Conference (WCNC).

[5] Lee J., Jo H., Oh J. (2023). Application of Drone LiDAR Survey for Evaluation of a Long-Term Consolidation Settlement of Large Land Reclamation. Appl. Sci..

[6] Shah S.A., Lakho G.M., Keerio H.A., Sattar M.N., Hussain G., Mehdi M., Vistro R.B., Mahmoud E.A., Elansary H.O. (2023). Application of drone surveillance for advance agriculture monitoring by Android application using convolution neural network. Agronomy.

[7] Famili A., Atalay T., Stavrou A., Wang H. Wi-Five: Optimal Placement of Wi-Fi Routers in 5G Networks for Indoor Drone Navigation. Proceedings of the 2023 IEEE 97th Vehicular Technology Conference (VTC2023-Spring).

[8] Fu X., Wei G., Yuan X., Liang Y., Bo Y. (2023). Efficient YOLOv7-Drone: An Enhanced Object Detection Approach for Drone Aerial Imagery. Drones.

[9] Zaitseva E., Levashenko V., Mukhamediev R., Brinzei N., Kovalenko A., Symagulov A. (2023). Review of Reliability Assessment Methods of Drone Swarm (Fleet) and a New Importance Evaluation Based Method of Drone Swarm Structure Analysis. Mathematics.

[10] Famili A., Stavrou A., Wang H., Park J.M.J. RAIL: Robust Acoustic Indoor Localization for Drones. Proceedings of the 2022 IEEE 95th Vehicular Technology Conference: (VTC2022-Spring).

[11] Khan M.A., Menouar H., Eldeeb A., Abu-Dayya A., Salim F.D. (2022). On the Detection of Unauthorized Drones—Techniques and Future Perspectives: A Review. IEEE Sens. J..

[12] Famili A., Stavrou A., Wang H., Park J.M.J. (2022). PILOT: High-Precision Indoor Localization for Autonomous Drones. IEEE Trans. Veh. Technol..

[13] Royo P., Asenjo A., Trujillo J., Cetin E., Barrado C. (2022). Enhancing Drones for Law Enforcement and Capacity Monitoring at Open Large Events. Drones.

[14] Gayathri Devi K., Yasoda K., Roy M.N. Automatic Firefighting System Using Unmanned Aerial Vehicle. Proceedings of the International Conference on Artificial Intelligence for Smart Community: AISC 2020.

[15] Bi Z., Guo X., Wang J., Qin S., Liu G. (2023). Deep reinforcement learning for truck-drone delivery problem. Drones.

[16] Eskandaripour H., Boldsaikhan E. (2023). Last-mile drone delivery: Past, present, and future. Drones.

[17] Larsen H.L., Møller-Lassesen K., Enevoldsen E.M.E., Madsen S.B., Obsen M.T., Povlsen P., Bruhn D., Pertoldi C., Pagh S. (2023). Drone with Mounted Thermal Infrared Cameras for Monitoring Terrestrial Mammals. Drones.

[18] Famili A., Stavrou A., Wang H., Park J.M. (2023). iDROP: Robust Localization for Indoor Navigation of Drones With Optimized Beacon Placement. IEEE Internet Things J..

[19] Zhao Y., Ju Z., Sun T., Dong F., Li J., Yang R., Fu Q., Lian C., Shan P. (2023). Tgc-yolov5: An enhanced yolov5 drone detection model based on transformer, gam & ca attention mechanism. Drones.

[20] Wang X., Yao F., Li A., Xu Z., Ding L., Yang X., Zhong G., Wang S. (2023). DroneNet: Rescue Drone-View Object Detection. Drones.

[21] Karpathakis S.F., Dix-Matthews B.P., Walsh S.M., McCann A.S., Gozzard D.R., Frost A.M., Gravestock C.T., Schediwy S.W. (2023). Ground-to-drone optical pulse position modulation demonstration as a testbed for lunar communications. Drones.

[22] George A., Koivumäki N., Hakala T., Suomalainen J., Honkavaara E. (2023). Visual-inertial odometry using high flying altitude drone datasets. Drones.

[23] Hou D., Su Q., Song Y., Yin Y. (2023). Research on drone fault detection based on failure mode databases. Drones.

[24] Famili A., Stavrou A., Wang H., Park J.M.J. SPIN: Sensor Placement for Indoor Navigation of Drones. Proceedings of the 2022 IEEE Latin-American Conference on Communications (LATINCOM).

[25] Ambesh R., Sarfraz A.B., Kapoor C., Joshi H., Patel H. Drone Detection using YOLOv4 and Amazon Rekognition. Proceedings of the 2022 International Conference on Applied Artificial Intelligence and Computing (ICAAIC).

[26] Tokosh J., Chen X. (2022). Delivery by Drone: Estimating Market Potential and Access to Consumers from Existing Amazon Infrastruture. Pap. Appl. Geogr..

[27] Campbell J.F. (2022). Will drones revolutionize home delivery?. Let’s get real… Patterns.

[28] Min H. (2023). Leveraging drone technology for last-mile deliveries in the e-tailing ecosystem. Sustainability.

[29] Weng Y.Y., Wu R.Y., Zheng Y.J. (2023). Cooperative truck–drone delivery path optimization under urban traffic restriction. Drones.

[30] AL-Dosari K., Hunaiti Z., Balachandran W. (2023). Systematic Review on Civilian Drones in Safety and Security Applications. Drones.

[31] Daud S.M.S.M., Yusof M.Y.P.M., Heo C.C., Khoo L.S., Singh M.K.C., Mahmood M.S., Nawawi H. (2022). Applications of drone in disaster management: A scoping review. Sci. Justice.

[32] Nguyen P., Truong H., Ravindranathan M., Nguyen A., Han R., Vu T. Matthan: Drone Presence Detection by Identifying Physical Signatures in the Drone’s RF Communication. Proceedings of the 15th Annual International Conference on Mobile Systems, Applications, and Services, MobiSys ’17.

[33] Aydin B., Singha S. (2023). Drone Detection Using YOLOv5. Eng.

[34] Seidaliyeva U., Ilipbayeva L., Taissariyeva K., Smailov N., Matson E.T. (2023). Advances and Challenges in Drone Detection and Classification Techniques: A State-of-the-Art Review. Sensors.

[35] Howell L.G., Allan B.M., Driscoll D.A., Ierodiaconou D., Doran T.A., Weston M.A. (2023). Attenuation of Responses of Waterbirds to Repeat Drone Surveys Involving a Sequence of Altitudes and Drone Types: A Case Study. Drones.

[36] Abbass M.A.B., Kang H.S. (2023). Drone elevation control based on python-unity integrated framework for reinforcement learning applications. Drones.

[37] Rábago J., Portuguez-Castro M. (2023). Use of Drone Photogrammetry as An Innovative, Competency-Based Architecture Teaching Process. Drones.

[38] Zhou Z., Yu X., Chen X. (2023). Object detection in drone video with temporal attention gated recurrent unit based on transformer. Drones.

[39] Iqbal U., Riaz M.Z.B., Zhao J., Barthelemy J., Perez P. (2023). Drones for Flood Monitoring, Mapping and Detection: A Bibliometric Review. Drones.

[40] Noetel D., Johannes W., Caris M., Hommes A., Stanko S. Detection of MAVs (Micro Aerial Vehicles) based on millimeter wave radar. Proceedings of the SPIE Security + Defence.

[41] Robie J., Famili A., Stavrou A. (2023). Revisiting the Spaceborne Illuminators of Opportunity for Airborne Object Tracking. Computer.

[42] Kolamunna H., Dahanayaka T., Li J., Seneviratne S., Thilakaratne K., Zomaya A.Y., Seneviratne A. (2021). DronePrint: Acoustic Signatures for Open-Set Drone Detection and Identification with Online Data. Proceedings of the ACM on Interactive, Mobile, Wearable and Ubiquitous Technologies.

[43] Zhang Y. RF-based Drone Detection using Machine Learning. Proceedings of the 2021 2nd International Conference on Computing and Data Science (CDS).

[44] Hammer M., Hebel M., Borgmann B., Laurenzis M., Arens M., Turner M.D., Kamerman G.W. (2018). Potential of LiDAR sensors for the detection of UAVs. Laser Radar Technology and Applications XXIII: Proceedings of SPIE Defense + Security, Orlando, FL, USA, 15–19 April 2018.

[45] Chahrour H., Dansereau R.M., Rajan S., Balaji B. (2021). Target Detection through Riemannian Geometric Approach with Application to Drone Detection. IEEE Access.

[46] Yang T., De Maio A., Zheng J., Su T., Carotenuto V., Aubry A. (2021). An Adaptive Radar Signal Processor for UAVs Detection With Super-Resolution Capabilities. IEEE Sens. J..

[47] Schneebeli M., Leuenberger A., Wabeke L., Kloke K., Kitching C., Siegenthaler U., Wellig P. Drone detection with a multistatic C-band radar. Proceedings of the 2021 21st International Radar Symposium (IRS).

[48] Yazici A., Baykal B. (2023). Detection and Localization of Drones in MIMO CW Radar. IEEE Trans. Aerosp. Electron. Syst..

[49] Fu R., Al-Absi M.A., Kim K.H., Lee Y.S., Al-Absi A.A., Lee H.J. (2021). Deep Learning-Based Drone Classification Using Radar Cross Section Signatures at mmWave Frequencies. IEEE Access.

[50] Semkin V., Yin M., Hu Y., Mezzavilla M., Rangan S. Drone Detection and Classification Based on Radar Cross Section Signatures. Proceedings of the 2020 International Symposium on Antennas and Propagation (ISAP).

[51] De Wit J.J., Gusland D., Trommel R.P. Radar Measurements for the Assessment of Features for Drone Characterization. Proceedings of the 2020 17th European Radar Conference (EuRAD).

[52] Zulkifli S., Balleri A. Design and Development of K-Band FMCW Radar for Nano-Drone Detection. Proceedings of the 2020 IEEE Radar Conference (RadarConf20).

[53] Griffin B., Balleri A., Baker C., Jahangir M. Optimal receiver placement in staring cooperative radar networks for detection of drones. Proceedings of the 2020 IEEE Radar Conference (RadarConf20).

[54] Morris P.J.B., Hari K.V.S. (2021). Detection and Localization of Unmanned Aircraft Systems Using Millimeter-Wave Automotive Radar Sensors. IEEE Sens. Lett..

[55] Maksymiuk R., Płotka M., Abratkiewicz K., Samczyński P. 5G Network-Based Passive Radar for Drone Detection. Proceedings of the 2023 24th International Radar Symposium (IRS).

[56] Lam I., Pant S., Manning M., Kubanski M., Fox P., Rajan S., Patnaik P., Balaji B. Time-Frequency Analysis using V-band Radar for Drone Detection and Classification. Proceedings of the 2023 IEEE International Instrumentation and Measurement Technology Conference (I2MTC).

[57] Mamat M.A.C., Aziz N.H.A. (2022). Drone Detection and Classification using Passive Forward Scattering Radar. Int. J. Integr. Eng..

[58] Gong J., Yan J., Li D., Kong D. (2022). Detection of Micro-Doppler Signals of Drones Using Radar Systems with Different Radar Dwell Times. Drones.

[59] Solomitckii D., Gapeyenko M., Semkin V., Andreev S., Koucheryavy Y. (2018). Technologies for Efficient Amateur Drone Detection in 5G Millimeter-Wave Cellular Infrastructure. IEEE Commun. Mag..

[60] Wang Y., Phelps T.A., Kibaroglu K., Sayginer M., Ma Q., Rebeiz G.M. 28 GHz 5G-Based Phased-Arrays for UAV Detection and Automotive Traffic-Monitoring Radars. Proceedings of the 2018 IEEE/MTT-S International Microwave Symposium—IMS.

[61] Chadwick A.D. Micro-drone detection using software-defined 3G passive radar. Proceedings of the International Conference on Radar Systems (Radar 2017).

[62] Yan J., Hu H., Gong J., Kong D., Li D. (2023). Exploring Radar Micro-Doppler Signatures for Recognition of Drone Types. Drones.

[63] Kapoulas I.K., Hatziefremidis A., Baldoukas A., Valamontes E.S., Statharas J. (2023). Small Fixed-Wing UAV Radar Cross-Section Signature Investigation and Detection and Classification of Distance Estimation Using Realistic Parameters of a Commercial Anti-Drone System. Drones.

[64] Gong J., Yan J., Hu H., Kong D., Li D. (2023). Improved Radar Detection of Small Drones Using Doppler Signal-to-Clutter Ratio (DSCR) Detector. Drones.

[65] Di Seglio M., Filippini F., Bongioanni C., Colone F. (2024). Comparing reference-free WiFi radar sensing approaches for monitoring people and drones. IET Radar Sonar Navig..

[66] Delamou M., Noubir G., Dang S., Amhoud E.M. (2023). An Efficient OFDM-Based Monostatic Radar Design for Multitarget Detection. IEEE Access.

[67] Rodriguez D., Rodrigues D.V.Q., Mishra A., Saed M.A., Li C. (2023). Quadrature and Single-Channel Low-Cost Monostatic Radar Based on a Novel 2-Port Transceiver Chain. IEEE Sens. J..

[68] Yuan H., Zhao S.Y., Chen Y.J., Luo Y., Liu Y.X., Zhang Y.P. (2023). Micro-Motion Parameters Estimation of Precession Cone Based on Monostatic Radar. IEEE Trans. Antennas Propag..

[69] Ding R., Wang Z., Jiang L., Liu Z., Zheng S. (2023). A target localisation method with monostatic radar via multi-observation data association. IET Radar Sonar Navig..

[70] Linder M., Strauch J., Schwarz D., Waldschmidt C. High Gain W-Band Lens Antenna for Monostatic Radar Applications: A System-Oriented Approach. Proceedings of the 2023 17th European Conference on Antennas and Propagation (EuCAP).

[71] Sakhnini A., Bourdoux A., Pollin S. (2023). Estimation of Array Locations, Orientations, Timing Offsets and Target Locations in Bistatic Radars. IEEE Trans. Radar Syst..

[72] Li H., Geng J., Xie J. (2023). Robust joint transmit and receive beamforming by sequential optimization for bistatic radar system. IET Radar Sonar Navig..

[73] Wu Y., Chen Z., Peng D. (2023). Target Detection of Passive Bistatic Radar under the Condition of Impure Reference Signal. Remote Sens..

[74] Xiong W., Lu Y., Song J., Chen X. (2023). A Two-Stage Track-before-Detect Method for Non-Cooperative Bistatic Radar Based on Deep Learning. Remote Sens..

[75] Santoro L., Nardello M., Fontanelli D., Brunelli D. (2023). UWB Bistatic Radar Sensor: Across Channels Evaluation. IEEE Sens. Lett..

[76] Robie J., Famili A., Stavrou A. Receiver Density Analysis for High Probability Detection of Forward Scattered Airborne Signals. Proceedings of the 2022 International Conference on Electrical, Computer and Energy Technologies (ICECET).

[77] Beasley P.J., Peters N., Horne C., Ritchie M.A. (2023). Global Navigation Satellite Systems disciplined oscillator synchronisation of multistatic radar. IET Radar Sonar Navig..

[78] Dhulashia D., Ritchie M. (2024). Multistatic radar distribution geometry effects on parameter estimation accuracy. IET Radar Sonar Navig..

[79] Randall M., Delacroix A., Ezell C., Kelderman E., Little S., Loeb A., Masson E., Watters W.A., Cloete R., White A. (2023). SkyWatch: A Passive Multistatic Radar Network for the Measurement of Object Position and Velocity. J. Astron. Instrum..

[80] Sruti S., Kumar A.A., Giridhar K. RCS-Based Imaging of Extended Targets for Classification in Multistatic Radar Systems. Proceedings of the 2023 IEEE Radar Conference (RadarConf23).

[81] da Graça Marto S., Díaz Riofrío S., Ilioudis C., Clemente C., Vasile M. (2023). Satellite manoeuvre detection with multistatic radar. J. Astronaut. Sci..

[82] Beasley P., Ritchie M., Griffiths H., Miceli W., Inggs M., Lewis S., Kahn B. Multistatic Radar Measurements of UAVs at X-band and L-band. Proceedings of the 2020 IEEE Radar Conference (RadarConf20).

[83] Shen X., Huang D. (2023). A Plane Wave Equivalent Model for Forward Scatter Shadow Ratio in Spherical Wave and Its Application in Shadow Profile Retrieval. IEEE Access.

[84] Sundaresan S., Surendar M., Ananthkumar T., Sureshkumar K., Prabhu J.S. (2023). Impact of wind farms on surveillance radar system: A realistic scenario in Palakkad gap region. J. Ambient. Intell. Humaniz. Comput..

[85] Shen X., Huang D. (2023). Forward Scatter Shadow Ratio: Concept and Its Application in Shadow Profile Retrieval. IEEE Access.

[86] Henry J.K., Narayanan R.M., Singla P. Design and processing of a self-mixing passive forward scatter radar fence for space debris tracking. Proceedings of the Sensors and Systems for Space Applications XVI.

[87] Yang R., Wang C.X., Huang J., Aggoune E.H.M., Hao Y. (2023). A Novel 6G ISAC Channel Model Combining Forward and Backward Scattering. IEEE Trans. Wirel. Commun..

[88] Oh B.S., Lin Z. (2021). Extraction of Global and Local Micro-Doppler Signature Features From FMCW Radar Returns for UAV Detection. IEEE Trans. Aerosp. Electron. Syst..

[89] Zhang Y.D., Xiang X., Li Y., Chen G. Enhanced Micro-Doppler Feature Analysis for Drone Detection. Proceedings of the 2021 IEEE Radar Conference (RadarConf21).

[90] Gannon Z., Tahmoush D. Measuring UAV Propeller Length using Micro-doppler Signatures. Proceedings of the 2020 IEEE International Radar Conference (RADAR).

[91] Dumitrescu C., Minea M., Costea I.M., Cosmin Chiva I., Semenescu A. (2020). Development of an Acoustic System for UAV Detection. Sensors.

[92] Fang J., Li Y., Ji P.N., Wang T. (2023). Drone Detection and Localization Using Enhanced Fiber-Optic Acoustic Sensor and Distributed Acoustic Sensing Technology. J. Light. Technol..

[93] Al-Emadi S., Al-Ali A., Al-Ali A. (2021). Audio-Based Drone Detection and Identification Using Deep Learning Techniques with Dataset Enhancement through Generative Adversarial Networks. Sensors.

[94] Salom I., Dimic G., Celebic V., Spasenovic M., Raickovic M., Mihajlovic M., Todorovic D. (2023). An Acoustic Camera for Use on UAVs. Sensors.

[95] Rascon C., Ruiz-Espitia O., Martinez-Carranza J. (2019). On the Use of the AIRA-UAS Corpus to Evaluate Audio Processing Algorithms in Unmanned Aerial Systems. Sensors.

[96] Basak S., Rajendran S., Pollin S., Scheers B. (2022). Combined RF-Based Drone Detection and Classification. IEEE Trans. Cogn. Commun. Netw..

[97] Allahham M.S., Khattab T., Mohamed A. Deep Learning for RF-Based Drone Detection and Identification: A Multi-Channel 1-D Convolutional Neural Networks Approach. Proceedings of the 2020 IEEE International Conference on Informatics, IoT, and Enabling Technologies (ICIoT).

[98] Medaiyese O.O., Syed A., Lauf A.P. Machine Learning Framework for RF-Based Drone Detection and Identification System. Proceedings of the 2021 2nd International Conference on Smart Cities, Automation & Intelligent Computing Systems (ICON-SONICS).

[99] Alam S.S., Chakma A., Rahman M.H., Bin Mofidul R., Alam M.M., Utama I.B.K.Y., Jang Y.M. (2023). RF-Enabled Deep-Learning-Assisted Drone Detection and Identification: An End-to-End Approach. Sensors.

[100] Flak P., Czyba R. (2023). RF Drone Detection System Based on a Distributed Sensor Grid With Remote Hardware-Accelerated Signal Processing. IEEE Access.

[101] Sazdić-Jotić B., Pokrajac I., Bajčetić J., Bondžulić B., Obradović D. (2022). Single and multiple drones detection and identification using RF based deep learning algorithm. Expert Syst. Appl..

[102] Kılıç R., Kumbasar N., Oral E.A., Ozbek I.Y. (2022). Drone classification using RF signal based spectral features. Eng. Sci. Technol. Int. J..

[103] Al-Emadi S., Al-Senaid F. Drone Detection Approach Based on Radio-Frequency Using Convolutional Neural Network. Proceedings of the 2020 IEEE International Conference on Informatics, IoT, and Enabling Technologies (ICIoT).

[104] Nemer I., Sheltami T., Ahmad I., Yasar A.U.H., Abdeen M.A.R. (2021). RF-Based UAV Detection and Identification Using Hierarchical Learning Approach. Sensors.

[105] Fang J., Zhou Z., Jin S., Wang L., Lu B., Qin Z. Exploring LoRa for Drone Detection. Proceedings of the IEEE INFOCOM 2022—IEEE Conference on Computer Communications Workshops (INFOCOM WKSHPS).

[106] Digulescu A., Despina-Stoian C., Popescu F., Stanescu D., Nastasiu D., Sburlan D. (2023). UWB Sensing for UAV and Human Comparative Movement Characterization. Sensors.

[107] Flak P. (2021). Drone Detection Sensor With Continuous 2.4 GHz ISM Band Coverage Based on Cost-Effective SDR Platform. IEEE Access.

[108] Mokhtari M., Bajcetic J., Sazdic-Jotic B., Pavlovic B. RF-based drone detection and classification system using convolutional neural network. Proceedings of the 2021 29th Telecommunications Forum (TELFOR).

[109] Lv H., Liu F., Yuan N. (2021). Drone presence detection by the drone’s RF communication. J. Phys. Conf. Ser..

[110] Chiper F.L., Martian A., Vladeanu C., Marghescu I., Craciunescu R., Fratu O. (2022). Drone detection and defense systems: Survey and a software-defined radio-based solution. Sensors.

[111] Sinha P., Yapici Y., Guvenc I., Turgut E., Gursoy M.C. RSS-Based Detection of Drones in the Presence of RF Interferers. Proceedings of the 2020 IEEE 17th Annual Consumer Communications & Networking Conference (CCNC).

[112] Basak S., Rajendran S., Pollin S., Scheers B. Drone classification from RF fingerprints using deep residual nets. Proceedings of the 2021 International Conference on Communication Systems & NETworkS (COMSNETS).

[113] Nie W., Han Z.C., Zhou M., Xie L.B., Jiang Q. (2021). UAV Detection and Identification Based on WiFi Signal and RF Fingerprint. IEEE Sens. J..

[114] Almubairik N.A., El-Alfy E.S.M. (2023). RF-Based Drone Detection with Deep Neural Network: Review and Case Study. Proceedings of the International Conference on Neural Information Processing.

[115] Morge-Rollet L., Le Jeune D., Le Roy F., Canaff C., Gautier R. (2022). Drone Detection and Classification Using Physical-Layer Protocol Statistical Fingerprint. Sensors.

[116] Nguyen P., Kim T., Miao J., Hesselius D., Kenneally E., Massey D., Frew E., Han R., Vu T. Towards RF-based localization of a drone and its controller. Proceedings of the 5th Workshop on Micro Aerial Vehicle Networks, Systems, and Applications.

[117] Yousaf J., Zia H., Alhalabi M., Yaghi M., Basmaji T., Shehhi E.A., Gad A., Alkhedher M., Ghazal M. (2022). Drone and Controller Detection and Localization: Trends and Challenges. Appl. Sci..

[118] Famili A., Foruhandeh M., Atalay T., Stavrou A., Wang H. GPS Spoofing Detection by Leveraging 5G Positioning Capabilities. Proceedings of the 2022 IEEE Latin-American Conference on Communications (LATINCOM).

[119] Himona G., Famili A., Stavrou A., Kovanis V., Kominis Y. Isochrons in tunable photonic oscillators and applications in precise positioning. Proceedings of the Physics and Simulation of Optoelectronic Devices XXXI.

[120] Sun Y., Wang W., Mottola L., Zhang J., Wang R., He Y. (2023). Indoor Drone Localization and Tracking Based on Acoustic Inertial Measurement. IEEE Trans. Mob. Comput..

[121] Famili A., Atalay T., Stavrou A., Wang H. Wi-Six: Precise Positioning in the Metaverse via Optimal Wi-Fi Router Deployment in 6G Networks. Proceedings of the 2023 IEEE International Conference on Metaverse Computing, Networking and Applications (MetaCom).

[122] Guvenc I., Ozdemir O., Yapici Y., Mehrpouyan H., Matolak D. Detection, localization, and tracking of unauthorized UAS and Jammers. Proceedings of the 2017 IEEE/AIAA 36th Digital Avionics Systems Conference (DASC).

[123] Famili A., Atalay T.O., Stavrou A., Wang H., Park J.M. (2023). OFDRA: Optimal Femtocell Deployment for Accurate Indoor Positioning of RIS-Mounted AVs. IEEE J. Sel. Areas Commun..

[124] Famili A., Slyusar V., Lee Y.H., Stavrou A. Vehicular Teamwork for Better Positioning. Proceedings of the 2023 IEEE International Conference on Systems, Man, and Cybernetics (SMC).

[125] Scholes S., Ruget A., Mora-Martín G., Zhu F., Gyongy I., Leach J. (2022). DroneSense: The Identification, Segmentation, and Orientation Detection of Drones via Neural Networks. IEEE Access.

[126] Dogru S., Marques L. (2022). Drone Detection Using Sparse Lidar Measurements. IEEE Robot. Autom. Lett..

[127] Chen Z., Miao Y., Tang D., Yang H., Pan W. (2022). Effect of LiDAR Receiver Field of View on UAV Detection. Photonics.

[128] Aldao E., Gonzalez-de Santos L.M., Gonzalez-Jorge H. (2022). Lidar Based Detect and Avoid System for UAV Navigation in UAM Corridors. Drones.

[129] Lv Y., Ai Z., Chen M., Gong X., Wang Y., Lu Z. (2022). High-Resolution Drone Detection Based on Background Difference and SAG-YOLOv5s. Sensors.

[130] Khan M.A., Menouar H., Khalid O.M., Abu-Dayya A. Unauthorized Drone Detection: Experiments and Prototypes. Proceedings of the 2022 IEEE International Conference on Industrial Technology (ICIT).

[131] Dudczyk J., Czyba R., Skrzypczyk K. (2022). Multi-Sensory Data Fusion in Terms of UAV Detection in 3D Space. Sensors.

[132] Laurenzis M., Hengy S., Hommes A., Kloeppel F., Shoykhetbrod A., Geibig T., Johannes W., Naz P., Christnacher F., Kadar I. (2017). Multi-sensor field trials for detection and tracking of multiple small unmanned aerial vehicles flying at low altitude. Signal Processing, Sensor/Information Fusion, and Target Recognition XXVI: Proceedings of SPIE Defense + Security, Anaheim, CA, USA, 9–13 April 2017.

[133] Laurenzis M., Hengy S., Hammer M., Hommes A., Johannes W., Giovanneschi F., Rassy O., Bacher E., Schertzer S., Poyet J.M., Kadar I. (2018). An adaptive sensing approach for the detection of small UAV: First investigation of static sensor network and moving sensor platform. Signal Processing, Sensor/Information Fusion, and Target Recognition XXVII: Proceedings of SPIE Defense + Security, Orlando, FL, USA, 15–19 April 2018.

[134] Wang Y., Phelps T., Rupakula B., Zihir S., Rebeiz G.M. 64 GHz 5G-Based Phased-Arrays for UAV Detection and Automotive Traffic-Monitoring Radars. Proceedings of the 2019 IEEE International Symposium on Phased Array System & Technology (PAST).

[135] Cao P. (2022). Cellular Base Station Imaging for UAV Detection. IEEE Access.

[136] Zhao J., Fu X., Yang Z., Xu F. (2019). Radar-assisted UAV detection and identification based on 5G in the Internet of Things. Wirel. Commun. Mob. Comput..

